# Fingernail Mineral Profiling as a Non‐Invasive Tool to Assess Dietary and Lifestyle Factors: Results From the Cross‐Sectional Fulda NutriNAIL Study

**DOI:** 10.1002/biof.70056

**Published:** 2025-11-14

**Authors:** Nina Sonntag, Lina Müller, Katja Plendl, Dustin Dewald, Jan‐Torsten Milde, Heike Hollenbach, Alexander Maxones, Tilman Kühn, Marc Birringer

**Affiliations:** ^1^ Department of Nutritional, Food and Consumer Sciences Fulda University of Applied Sciences Fulda Germany; ^2^ Department of Computer Sciences Fulda University of Applied Sciences Fulda Germany; ^3^ Wissenschaftliches Zentrum für Ernährung, Lebensmittel Und Nachhaltige Versorgungssysteme (ELVe) Fulda University of Applied Sciences Fulda Germany; ^4^ Public Health Zentrum Fulda (PHZF) Fulda University of Applied Sciences Fulda Germany

**Keywords:** dietary patterns, fingernail mineral analysis, ICP‐MS, nutritional epidemiology, selenium

## Abstract

In the past, fingernail and toenail analyses were mainly used for toxicological investigations on heavy metals and only a few studies used mineral content as a biomarker for assessing lifestyle or dietary habits. The Fulda NutriNAIL study examined associations between lifestyle, dietary habits, supplement use, and fingernail mineral composition in 184 participants (18–81 years) from Fulda, Germany. Mineral concentrations were quantified via Inductively Coupled Plasma Mass Spectrometry (ICP‐MS), and questionnaire data were analyzed for lifestyle correlations. Positive mineral‐to‐mineral correlations were observed between sodium (Na) and potassium (K) (*r* = 0.89), calcium (Ca) and magnesium (Mg) (*r* = 0.57), and iron (Fe) and cobalt (Co) (*r* = 0.66). We detected 21% higher selenium levels in the fingernails of selenium supplement users than for non‐users. Omnivores showed higher Se levels than vegans and slightly higher levels than vegetarians and participants with thyroid disorders had significantly higher nail selenium levels. In addition, nail surface features were linked to specific mineral differences: potassium in brittle nails, reduced sodium and potassium in longitudinal ridges, and reduced chromium (Cr) in white spots. These findings demonstrate that fingernail mineral profiling is a non‐invasive method for assessing lifestyle‐ and diet‐related micronutrient status.

## Graphical abstract

ICP‐MS profiling of fingernails and assessment of the dietary habits and lifestyle of 184 adults revealed correlations between Na–K, Ca–Mg and Fe–Co, as well as significant differences in selenium content based on the participants' diets. In addition, nail surface characteristics were associated with mineral differences. The study supports non‐invasive health monitoring.

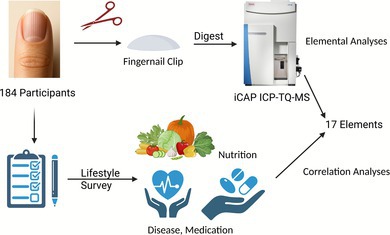

## 1 Introduction

Hair, toenails and fingernails consist mainly of fibrous α‐keratin proteins embedded in a matrix of lipids and other structural proteins. In addition, minerals, trace elements and toxic metals can be deposited. Studies on the mineral content of toenails are part of human biomonitoring efforts, investigating the environmental burden of heavy metals and other toxins. A systematic review of Gutiérrez et al. evaluates the potential of toenail clippings as biomarkers for long‐term exposure to essential trace metals, with a focus on their validity, intraindividual variability, and correlation with other biomarkers [1]. The authors summarize that toenail concentrations of selenium (Se), manganese (Mn), and zinc (Zn) appear to reflect long‐term exposure fairly reliably; however, the variability among studies and the lack of standardization in sample collection and analysis limit their broader validation in epidemiological research. Dietary patterns, chronic diseases, environmental factors and medication influence the mineral composition and morphology of fingernails [2, 3, 4, 5, 6]. However, few studies focused on the association between sociodemographic data, lifestyle and health‐related patterns with the mineral content of fingernails. A study of Cai et al. [7] investigates the relationship between diet and longevity by analyzing elemental profiles in the fingernails of healthy individuals over 80 years old from a “longevity region” in China. The researchers identified four key elements—chromium, iron, manganese and cobalt—that were significantly higher in individuals from the longevity region compared to a control group. The study suggests that long‐term dietary habits and environmental exposure play a crucial role in maintaining elemental homeostasis, potentially contributing to longevity. Investigations of the trace element selenium in toe‐ and fingernails have a long history. Its concentration correlates with selenium plasma levels and with dietary habits, sex, and lifestyle factors such as smoking, alcohol consumption or selenium supplementation [8, 9, 10, 11].

The Fulda NutriNAIL study aims to investigate the influence of dietary habits and lifestyles on the mineral composition of fingernails. It will examine the relationships between individual mineral content and dietary patterns, as well as diseases, medication use and physical activity. This non‐invasive analysis method will support AI‐based pattern recognition of fingernail surfaces in the future. We here describe the study design of the Fulda NutriNAIL study, including a nutrition and lifestyle survey, study participants' characteristics, data acquisition and the determination of the mineral composition of the fingernail samples by Inductively Coupled Plasma Mass Spectrometry (ICP‐MS). In addition, first data on associations between dietary and lifestyle factors and selenium content of the nails are presented.

## 2 Experimental Procedures

### 2.1 Study Design

The NutriNAIL trial is a German cross‐sectional study approved by the local Ethics Committee (Fulda University of Applied Sciences, EK240519‐Birringer‐end). A written declaration of consent was obtained from all participants, which was supplemented by written and verbal information. The trial has been registered in the German Clinical Trials Register (reference number: DRKS00034566).

The participants in the study were recruited from May 2024 onwards, through a variety of methods including announcements on the university's social media accounts and website (both public and intranet), posters, and a newspaper announcement. Short fingernails (< 1 mm distal edge of nail plate) and age below 18 years were exclusion criteria. No further exclusion criteria were applied.

### 2.2 Data Collection

Data collection has been performed by an online survey which was available in German and English languages. Furthermore, fingernails have been collected from each participant. To collect data, study participants went through three stations: the picture recording station for the fingernail surface pattern, the station for cutting the nails and an electronic device station for answering the online questionnaire. Fingernails were clipped with stainless steel scissors or clippers and collected in small zipper plastic bags. The identity of the participants was anonymized by QR code labeling of the zipper bag. This QR code was scanned by the participants' smartphones or provided device to access the survey software. Later, the QR code was used to label the sample sets for mineral content analyses.

An online questionnaire on sociodemographic and behavioral characteristics, food intake, supplement use as well as questions on health status was administered to the participants. The questionnaire was implemented in SoSci [12]. The questionnaire distinguished between the nutritional forms “omnivore, pescatarian, ovo‐lacto‐vegetarian, lacto‐vegetarian, ovo‐vegetarian, vegan, and others”. We assessed the dietary habits and food patterns through a semi‐quantitative food frequency questionnaire according to the German National Nutrition Survey II [13] that asked about usual consumption of food groups eaten during the last 4 weeks. For each food category, a scale range of “never; less than once a month; 1–3 times a month; 1–3 times a week; 4–6 times a week; once a day and several times a day” was used.

Food items were compiled and categorized according to the German Nutrition Society (DGE) guidelines, evidence from published studies on dietary habits and mineral content of nails as well as nutrient‐rich foods with potential correlation with mineral content [14]. The semiquantitative food frequency questionnaire (FFQ) along with the food items and questions on health status and lifestyle including weight and height can be found in the Supporting Information. Our FFQ has not been validated before the study.

In brief, we asked the participants about chronic diseases and diagnosed nutritional deficiencies, their regular use of medication (free input option), any diagnosed nutrient deficiency (which and current status), their physical activity level (PAL), weekly exercise (and location i.e., indoor or outside), smoking habits, abnormalities of fingernail structure, fingernail hygiene and the use of nail polish (also metal‐containing polish). Sociodemographic questions include biological gender, age and education.

To investigate the intra‐individual changes in mineral contents of fingernails along a 5.5‐month period, a female volunteer (55 years) collected fingernails every 2 weeks. During that time frame, no changes in lifestyle have been (self‐)reported.

### 2.3 Mineral Content Analyses

#### 2.3.1 Fingernail Collection and Cleaning

Collected nail samples were collected in zipper plastic bags and stored at room temperature until analysis. The IAEA cleaning procedure [15] for hair and nails was adapted. In brief, fingernail samples (approx. 30 mg) were sonicated in acetone (Carl Roth GmbH), water and acetone for 10 min each. The nails were dried in vacuum (vac speed) at 45°C for 60 min until their weight remained constant and were weighed precisely before microwave (MLS Mikrowellen‐Labor‐Systeme GmbH) assisted digestion.

#### 2.3.2 Determination of Minerals in Fingernail Clippings by ICP‐TQ‐MS

Fingernail clippings were poured into a 5.8 mL quartz vial containing a solution of 1 mL of 2% nitric acid (VWR Chemicals), 500 μL nitric acid (67%–69%), 150 μL hydrogen peroxide (30% H_2_O_2_, Suprapur, Sigma Aldrich) and 150 μL internal standard solution (Rhenium 200 μg/L). A standard series of multielement standards (multielement standard 36, molybdenum (Mo), sodium, iron, calcium and zinc were obtained from VWR Chemicals) is produced for external calibration. Single element standards for sulfur, phosphorus (P) and magnesium (Inorganic Ventures) and potassium and rhenium (internal standard) (ThermoFischer Scientific) were used. Within this standard series, three concentration ranges are covered: 0–20 μg/L (low), 0–620 μg/L (medium) and 0–75 mg/L (only sulfur). Ultrapure water was generated in house with AriumComfort II (Sartorius). The microwave power was increased from 700 W to 1000 W during 40 min in an ethos.laboratory Microwave. After cooling of the reaction mixture, the clear solution was transferred into a metal free plastic tube and the reaction vial was rinsed twice with 2% nitric acid. Samples were measured with an iCAP TQ‐ICP‐MS (Thermo Fisher) equipped with an auto‐sampler ASX 280 (Teledyne). The following elements were measured in the fingernail digest: Lithium (Li), B, Na, Mg, (P), S, K, Ca, Cr, Mn, Mo, Fe Co, Ni, Cu, Zn, and Se. The integrated ICP‐MS software suggested the mode (on‐mass or mass shift) with the lowest known interferences. For example, Ca is measured as ^44^Ca^+^, Se as ^80^Se^16^O^+^ in DRC (dynamic reaction cell) mode, as well as Cr as ^52^Cr^16^O^+^ and S in DRC mode. To ensure the quality of the analysis data, the certified reference material (CRM) “String Beans” (LGC Standards, WEPAL‐IPE‐192) was analyzed in parallel with the samples. This reference material was used to validate the measurement results and ensure the reliability and accuracy of the data collected. The recoveries of the elemental measurements for CRM were within the accepted range of 80%–120%. Limit of detection (LOD) and limit of quantification (LOQ) were calculated for each element according to the following equations: LOD = 3.3 × *σ*/*S* and LOQ = 10 × *σ*/*S*, where *σ* is the standard deviation of response and S the slope of the calibration curve [16].

### 2.4 Statistical Analysis

A total of 202 persons participated in the study. However, 18 subjects had to be excluded from the analysis (11 cases with technical problems during the study, 4 cases with missing nail analysis data, 3 cases with incomplete/unfinished surveys). Missing data was assumed to be missing completely at random (MCAR) due to the above reasons and treated using the listwise deletion method, thus performing a per protocol analysis. Free entry options were analyzed and corrected where possible. For the statistical evaluation of the FFQ and to gain higher statistical power between groups, we decided to transform the following scales for all responses. We retained “never, rarely, several times a month” and combined “1–3 times a week and 4–6 times a week” into ‘weekly’ and “once a day and several times a day” into “daily”. Descriptive statistics were conducted for all parameters and expressed as mean and standard deviation (SD). Medians and interquartile ranges were obtained for key characteristics as the basis of box plots. Spearman's coefficients were computed to assess correlations between metric variables such as inter‐element correlations. Log(e)‐relative differences and 95% confidence intervals (CIs) were obtained to assess relative differences in fingernail mineral concentrations across strata of the study population. Relative differences were considered statistically significant whenever the 95% confidence interval did not include the reference category (i.e., entirely above or below zero). All analyses were conducted using IBM SPSS Statistics (Version 29, IBM Corporation, Armonk, NY, USA) and R Studio (RStudio, PBC, Boston, MA).

## 3 Results

### 3.1 Study Population Characteristics

The study population (77.7% women) had a mean age of 38.5 years (18(min)– 83(max)). More than 40% of the individuals possess a qualification that grants access to higher education in Germany. Mean Body Mass Index (BMI) was determined as 23.4 kg/m^2^ (15.8(min)– 41.8(max)). In total, 62% of participants indicated they are omnivores, 11% of the participants were vegan, 20% vegetarian and 7% pescatarians (Table 1). The Physical Activity Level (PAL) showed a moderate level of 1.6–1.7 in 65% of participants. More than 80% of participants stated that they exercise 1–2 times a week. We found that 75% of the participants took one or more dietary supplements with vitamin D as the most used (*n* = 96, 52%), followed by magnesium (*n* = 77, 41.8%) and vitamin B_12_ (*n* = 60, 32.6%). Regular alcohol consumption (min. 1–3× week) was found for 18% of participants. In total, 5.4% of the participants were smoking regularly.

**TABLE 1:** Characteristics of study population. Socioeconomic and lifestyle data.

Variables	Total (*n* = 184)	Variables	Total (*n* = 184)
Age (years)		Physical activity level (PAL)	
Mean (SD)	38.51 (18.82)	1.2–1.3 (*n*)	5 (2.7%)
Sex (m/f) (*n*)	41/143 (22.3%/77.7%)	1.4–1.5 (*n*)	32 (17.4%)
Education		1.6–1.7 (*n*)	120 (65.2%)
Lower secondary school certificate (*n*)	5 (2.7%)	1.8–1.9 (*n*)	19 (10.3%)
Intermediate secondary school certificate (*n*)	20 (10.9%)	2.0–2.4 (*n*)	8 (4.4%)
General or subject‐specific higher education entrance qualification (*n*)	75 (40.8%)	Regular exercise	
Bachelor's degree (*n*)	32 (17.4%)	5× or more a week (*n*)	20 (10.9%)
Master's degree/Doctorate (*n*)	31 (16.8%)	3–4× a week (*n*)	51 (27.7%)
Completed vocational training/Master craftsman certificate(*n*)	11 (6.0%)	1–2× a week (*n*)	87 (47.3%)
Other (*n*)	10 (5.4%)	Never (*n*)	26 (14.1%)
BMI (kg/m^2^)		Drinking	
Mean (range)	23.4 (15.8–41.8)	Beer (regular/≤ 1–3× month/never) (*n*)	33/108/42
Diet		Wine (regular/≤ 1–3× month/never) (*n*)	33/109/43
Omnivore (*n*)	115 (62.5%)	Spirits (regular/≤ 1–3× month/never) (*n*)	11/114/58
Pescatarian (*n*)	13 (7.0%)	Smoking	
Vegetarian (*n*)	36 (19.6%)	(regular/≤ 1–3× month/never) (*n*)	10/11/163
Vegan (*n*)	20 (10.9%)		
Self‐reported use of supplements^a^ (*n*)	138 (75%)		
Biotin (*n*)	11 (6%)		
Calcium (*n*)	21 (11.4%)		
Iron (*n*)	38 (20.7%)		
Iodine (*n*)	18 (9.8%)		
Magnesium (*n*)	77 (41.8%)		
Selenium (*n*)	20 (10.9%)		
Zink (*n*)	26 (14.1%)		
Vitamin B_12_ (*n*)	60 (32.6%)		
Vitamin D (*n*)	96 (52.2%)		
Other B‐vitamins^b^ (*n*)	14 (7.6%)		
Omega‐3 fatty acids (*n*)	11 (6.0%)		
Others^c^ (*n*)	13 (7.1%)		

*Note:* SD or % are depicted in brackets.

Abbreviations: BMI, body mass index; SD, standard deviations.

^a^ Multiple choices.

^b^ B1, B2, B6, Folic acid, B‐complex.

^c^ Amino acids, Cu, potassium, curcumin, Q10, GABA, choline, Vit C/A/E/K, multi supplement.

Chronic allergies (allergic rhinitis, hay fever, contact allergy) (*n* = 37, 20.1%), thyroid disease (*n* = 35, 19%), dyslipidemia (*n* = 21, 11.4%), high blood pressure (*n* = 20, 10.9%), joint disease (*n* = 16, 8.7%) and food allergies (*n* = 12, 6.5%) were the most frequently self‐reported diseases (*n* > 10 participants). Seventy‐seven participants (41.8%) regularly take medication (Table 2).

**TABLE 2:** Medical history of the study participants.

Self‐reported disease (*n* > 10)	Total (*n* = 184)	Self‐reported fingernail texture	Total (*n* = 184)	Diagnosed nutrient deficiency	Total (*n* = 184)
Allergic rhinitis, hay fever, contact allergy (*n*)	37 (20.1%)	Brittle (*n*)	20 (11%)	Yes (*n*)	82 (44.6%)
Thyroid disease (*n*)	35 (19%)	Thin (*n*)	8 (4.3%)	No (*n*)	88 (47.8%)
Dyslipidemia^a^ (*n*)	21 (11.4%)	White spots (*n*)	33 (17.9%)	I don't know (*n*)	14 (7.6%)
High blood pressure (*n*)	20 (10.9%)	Discoloration (*n*)	1 (0.5%)	Current (*n*)	14 (7.6%)
Neurodermatitis (*n*)	16 (8.7%)	Longitudinal ridges (*n*)	39 (21.2%)	Nutrient	
Joint disease^b^ (*n*)	16 (8.7%)	Others (*n*)	25 (13.6%)	Iron (ever)	55 (29.9%)
				Iron (current) (*n*)	11 (6%)
Food allergies (*n*)	12 (6.5%)	None (*n*)	58 (31.5%)	Vitamin D (ever)	32 (17.4%)
				Vitamin D (current) (*n*)	7 (3.8%)
Regular use of one or more medications (*n*)	77 (41.8%)			Vitamin B_12_ (ever)	11 (6%)
				Vitamin B_12_ (current) (*n*)	4 (2.2%)

^a^ High cholesterol and/or high plasma triglycerides.

^b^ Arthritis, arthrosis, rheumatoid arthritis.

### 3.2 Dietary Intake

An example of the food frequency distribution among the participants along with the selenium content of fingernails is shown in Table S1 (supplement). The results are presented without the cases involving “selenium supplement use.” Descriptive statistics indicated slightly higher selenium levels with greater consumption of fish and meat. In addition to food groups, participants were asked about the beverages they mainly use to meet their fluid requirements (multiple selections were possible). Tap water was the most frequently selected beverage (*n* = 127, 69%), followed by commercial mineral water (*n* = 57, 31%), tea (*n* = 57, 31%), coffee (*n* = 53, 28.8%), and sweetened beverages (*n* = 10, 5.4%). Most people used iodized table salt (*n* = 93, 50.5%). The purchase of organic food was reported by most people as “frequent” to “almost always” (*n* = 111, 60.3%).

Out of 170 participants, 82 (44.6%) were diagnosed with a micronutrient deficiency. Iron deficiency (*n* = 55, 29.9%) and vitamin D deficiency (*n* = 32, 17.4%) were mentioned most frequently, followed by vitamin B_12_ (*n* = 11, 6%). A current deficiency was reported by 14 (7.6%) participants (Table 2).

### 3.3 Characteristics of Fingernails

Questions were asked about fingernail care, the use of nail polish and nail diseases. Of particular interest were the individual characteristics of the nail structure. We asked about nail stability, longitudinal and transverse ridges and white spots within the nail structure since special features of the nail surface can provide indications of defects or diseases and will be essential for the AI learning algorithms later on (Table 2). The study participants named longitudinal ridges as the most common structural feature (*n* = 39, 21%), followed by white spots (*n* = 33, 18%) and brittle (*n* = 20, 11%) or thin (*n* = 8, 4.3%) fingernails.

### 3.4 Distribution of Minerals and Inter‐Individual Variations

The mineral content of fingernail samples is displayed as mean value with standard deviation (SD) and median in Table 3. The coefficient of variation (CV%) between individuals was 223% for boron and 260% for cobalt followed by nickel (175%) and molybdenum (143%). Lowest variations were found for sulfur (16.9%), selenium (20.5%), magnesium (35%), zinc (35.1%), and phosphorous (39.4%).

**TABLE 3:** Geometric means and median of fingernail mineral content including an intra‐individual sample set for a 5.5 month follow‐up.

Elements	LOQ	Mean^a^	SD (%CV)	Min	Max	Median^a^	5.5 months intra‐individual mineral content^b^ (SD) (%CV)
Boron (μg/g)	1.3	0.57	1.27 (222.8%)	<LOD	11.63	0.24	0.12 (0.04) (33.3%)
Calcium (μg/g)	123.2	608.96	373.18 (61.3%)	<LOD	2242.65	599.71	579.4 (58.2) (10%)
Chromium (μg/g)	0.7	0.21	0.21 (100%)	<LOD	1.52	0.15	0.17 (0.11) (65%)
Cobalt (μg/g)	0.04	0.05	0.13 (260%)	<LOD	1.09	0.01	0.01 (0.004) (36.4%)
Copper (μg/g)	1.4	7.50	5.53 (73.7%)	<LOD	50.63	6.08	5.6 (0.63) (11.2%)
Iron (μg/g)	23.4	18.88	19.67 (104.2%)	<LOD	135.65	13.34	13.1 (5.7) (43.6%)
Lithium (μg/g)	0.09	0.04	0.04 (100%)	<LOD	0.27	0.03	<LOD
Magnesium (μg/g)	17.1	89.38	31.24 (35%)	23.94	280.74	86.13	74.5 (2.2) (2.9%)
Manganese (μg/g)	0.36	0.24	0.33 (137.5%)	<LOD	1.66	0.18	0.13 (0.06) (43.4%)
Molybdenum (μg/g)	0.06	0.014	0.02 (142.9%)	<LOD	0.18	0.009	0.009 (0.007) (78%)
Nickel (μg/g)	0.32	0.69	1.21 (175.4%)	<LOD	8.48	0.31	0.67 (0.41) (61.5%)
Phosphorous (μg/g)	42.8	239.43	94.26 (39.4%)	<LOD	545.98	219.53	191.0 (10.2) (5.3%)
Potassium (μg/g)	25.3	160.65	134.00 (83.4%)	<LOD	833.75	133.26	56.7 (13.2) (23.3%)
Selenium (μg/g)	0.19	0.44	0.09 (20.45%)	0.22	0.71	0.42	0.55 (0.032) (5.8%)
Sodium (μg/g)	27.9	224.02	168.86 (75.4%)	<LOD	919.05	176.50	88.4 (16.3) (18.4%)
Sulfur (mg/g)	8.9	29.69	5.00 (16.9%)	<LOD	49.15	29.30	29.1 (0.83) (2.9%)
Zinc (μg/g)	31.0	125.54	44.01 (35.1%)	58.27	404.18	113.94	173.3 (5.3) (3.1%)

^a^ Fingernail mineral content was determined for all participants (*n* = 184).

^b^ Data were recorded from a female every 2 weeks during 5.5 months (mean of *n* = 11 samples, ± SD). Standard deviation (SD); percent coefficient of variation (%CV).

### 3.5 Intra‐Individual Variations During a 5.5 Months Period

In total, 11 samples were investigated with the highest intra‐individual variations for molybdenum content (CV 78%) followed by chromium (65%) and nickel (61.5%). Lowest variations were seen in the contents of magnesium (2.9%), sulfur (2.9%), zinc (3.1%), phosphorous (5.3%), selenium (5.8%) and calcium (10%).

### 3.6 Inter‐Mineral Correlation Analysis

**FIGURE 1:**
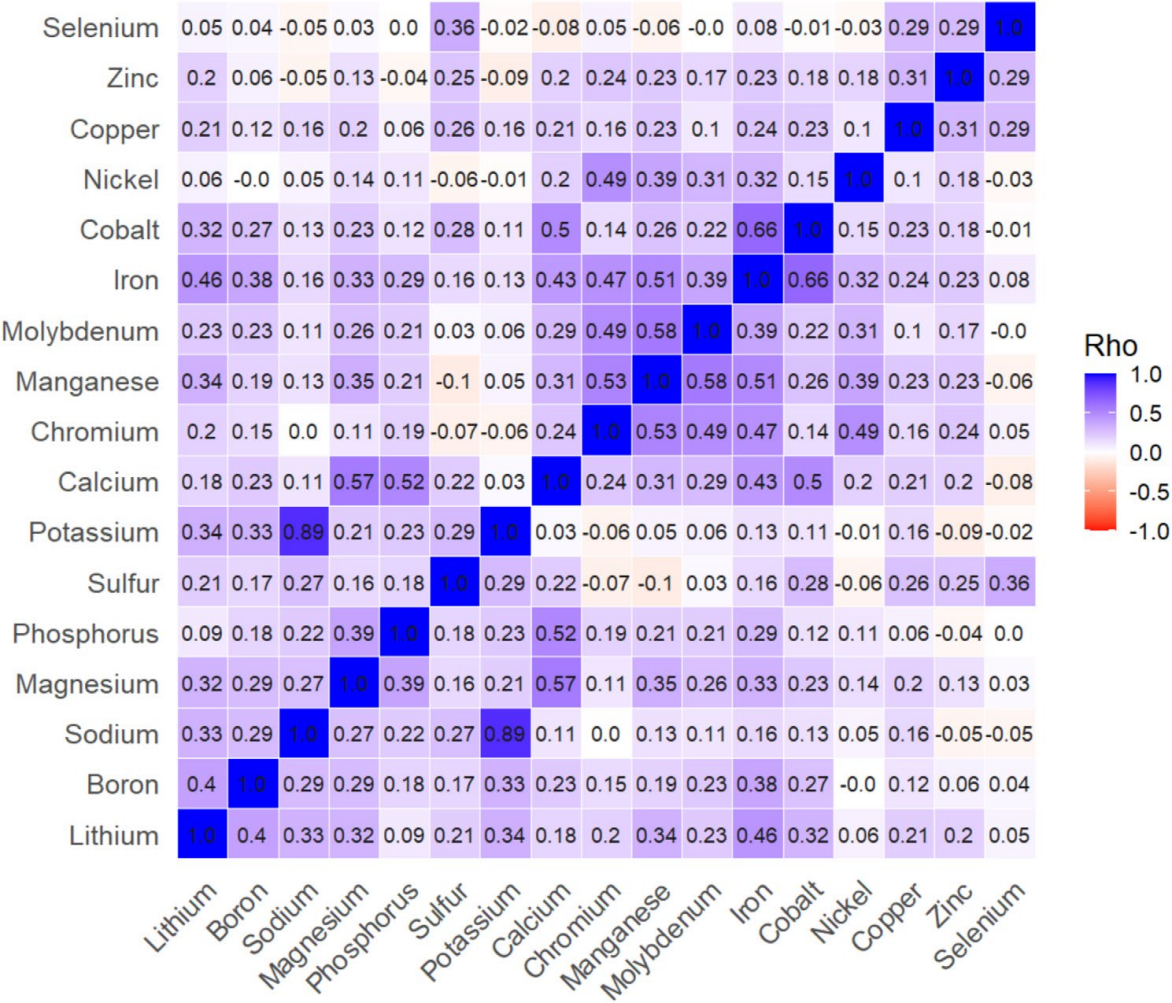
Spearman's rank correlation coefficient (Rho) of investigated elements based on the data of all 184 participants. Blue colored squares show positive correlations whereas red colored squares show invers correlations. Color intensity follows the strength of correlation.

Inter‐element correlation analysis was performed based on Spearman correlation. A heat map (Figure 1) shows the correlations between the individual elements with the diagonal as the defined value 1. A strong positive correlation was determined for the elements sodium and potassium (*r* = 0.89), while moderate correlations were observed for the element pairs calcium and magnesium (*r* = 0.57) and calcium and phosphorus (*r* = 0.52). Manganese shows modest positive correlations with molybdenum (*r* = 0.58), iron (*r* = 0.51) and chromium (*r* = 0.53). Furthermore, the values for iron and cobalt (*r* = 0.66) positively correlate. Selenium shows weaker positive correlations to sulfur (*r* = 0.36), zinc (*r* = 0.29), and copper (*r* = 0.29).

### 3.7 Fingernail Structure and White Spots Are Associated With Specific Element Concentrations

As described above 126 participants reported peculiarities in their fingernail structure or coloration. Brittle fingernails (*n* = 29, 15.8%) were associated with lower potassium concentrations (−30.3%). Longitudinal ridges (*n* = 51, 27.7%) of the fingernails were associated with lower potassium (−29.0%) sodium (−22.4%) concentrations. The nail analyses of participants with white spots (*n* = 39, 21.2%) showed a significantly lower chromium content (−29.1%) (Table 4).

**TABLE 4:** Mineral levels (μg/g) and finger nail quality.

Mineral	Nail quality parameter	*N*	Median (IQR)	Relative differences (95% CI)^a^
Potassium	Normal nails	155	141.5 (122.1)	Ref.
	Brittle nails	29	73.1 (109.8)	−30.3 (−49.4–4.0)
Potassium	No longitudinal ridges	133	145.9 (139.1)	Ref.
	Longitudinal ridges	51	104.8 (103.4)	−29.0 (−45.2–7.9)
Sodium	No longitudinal ridges	133	201.8 (205.2)	Ref.
	Longitudinal ridges	51	145.1 (140.2)	−22.4 (−39.5–0.4)
Chromium	No white spots	145	0.17 (0.18)	Ref.
	White spots	39	0.14 (0.17)	−29.1 (−49.4–0.5)

^a^ Calculated as log(e) relative differences.

### 3.8 Fingernail Selenium Content and Association With Lifestyle Factors

**FIGURE 2:**
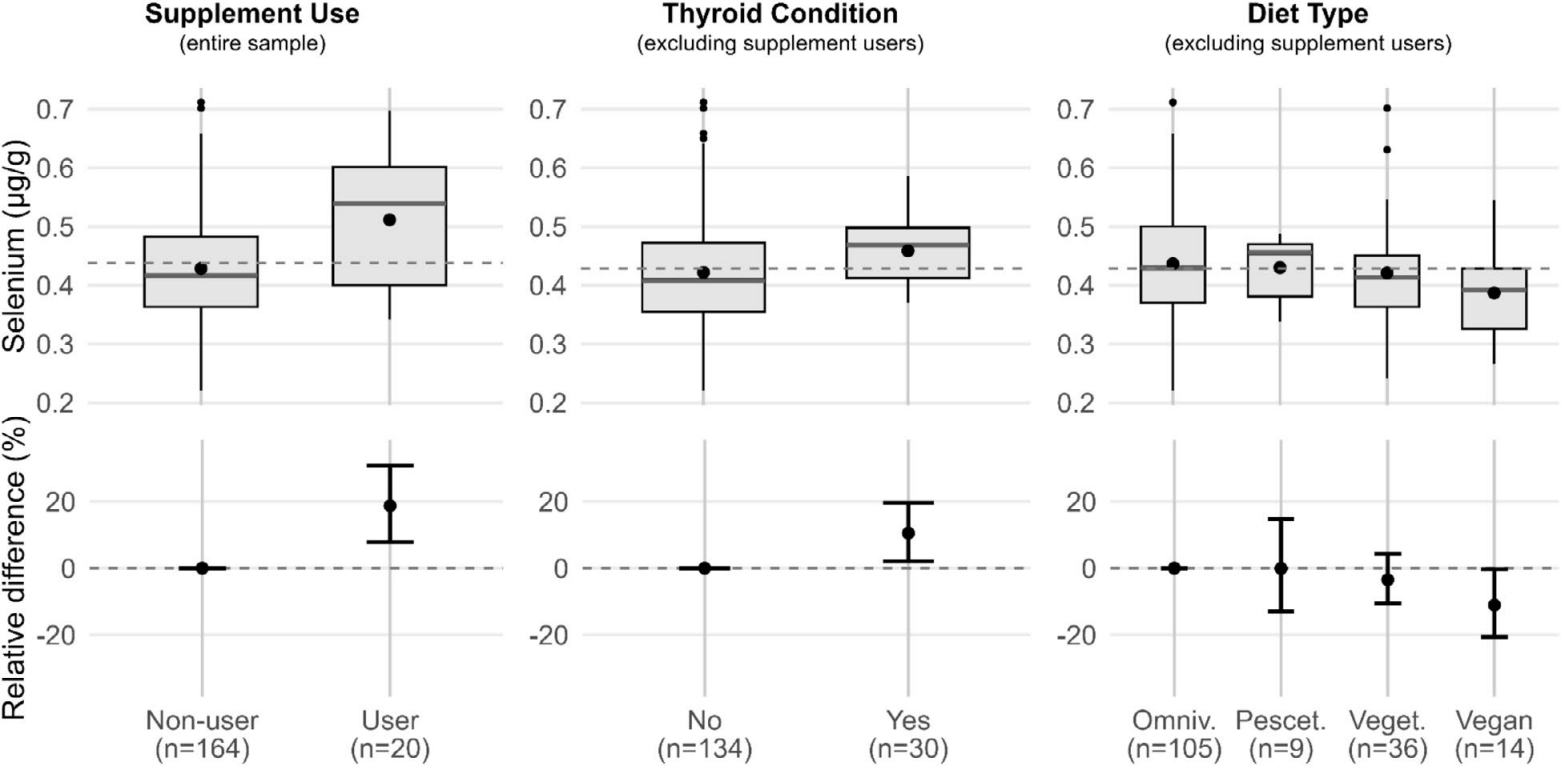
Selenium levels across strata of the study population. The upper panel shows box plots of absolute selenium concentrations across different subgroups of the study population. Dashed reference lines indicate overall mean levels. The big dots indicate mean values within subgroups. The lower panel depicts relative differences across groups compared to the respective reference groups (non‐users of supplements, individuals without thyroid condition or omnivorous individuals). Log(e) relative differences were obtained.

We found a statistically significant difference in selenium content of fingernails between supplement users (*n* = 20) and non‐users (Figure 2A and Table 5). The mean value of supplement users is 18.7% higher than that of non‐users (Table 2). We therefore decided to restrict analyses on further key covariates to non‐users of selenium supplements. Men had significantly lower fingernail selenium concentrations than women (−8.6%). No differences in concentrations across strata of age were observed. Having a thyroid condition was associated with 10.5% higher selenium concentrations (Figure 2B). Participants, who followed a vegan diet, had 11.1% lower fingernail selenium (Figure 2C). Regular exercise, PAL or BMI were not associated with Se content of nails (data not shown).

**TABLE 5:** Nail selenium levels (μg/g) across selected strata of the study population.

		*N*	Median (IQR)	Relative differences(95% CI)^a^
Supplement use	Non‐users	164	0.42 (0.12)	Reference
	Users	20	0.54 (0.20)	18.7 (7.8–30.8)
Sex^b^	Female	130	0.42 (0.1)	Reference
	Male	34	0.38 (0.18)	−8.6 (−15.3–1.3)
Age Strata^b^	18–40 years	107	0.42 (0.13)	Reference
	40–60 years	23	0.44 (0.1)	3.9 (−5.3–14)
	> 60 years	34	0.41 (0.1)	−1.7 (−9.2–6.4)
Thyroid condition^b^	No	134	0.41 (0.12)	Reference
	Yes	30	0.47 (0.09)	10.5 (2–19.7)
Diet type^b^	Omnivorous	105	0.43 (0.13)	Reference
	Pescetarian	9	0.46 (0.09)	−0.1 (−13–14.8)
	Vegetarian	36	0.41 (0.09)	−3.5 (−10.6–4.3)
	Vegan	14	0.39 (0.10)	−11.1 (−20.6–0.4)

^a^ Calculated as log(e) relative differences.

^b^ Only among non‐users of selenium supplements.

## 4 Discussion

This study, involving 184 participants, evaluates the relationship between lifestyle factors, health aspects and dietary habits and the mineral composition of fingernails. We could show that there are specific inter‐element correlations and that the Se content of nails may depend on diet type, with lower levels among vegans. With regard to the latter finding it should be noted that the proportion of vegetarians and vegans is disproportionately represented at 19.6% and 10.9%, respectively, compared to the average number of these individuals in Germany with 11.4% and 2.1% [17]. Another distinctive feature of our study population was the high number of supplement users, which at 75% is significantly higher than the German average of 51% [18]. The fact that the German Nutrition Society recommends the use of dietary supplements for a plant‐based diet could explain their predominant use [19].

Our study shows that the mineral content in nails varied considerably between the participants (CV 16.9%– 260%). In contrast, the intra‐individual content (only 1 person) was relatively stable (CV 2.9%–78%) during a 5–6‐month study period. The mean variation coefficient between the participants is 5.4 (1.8–12.1) times higher than within the measurements of one participant during the time period mentioned. It is known that the iron, chromium, manganese, cobalt, molybdenum levels in toenails underlie seasonal fluctuations (higher in summer than in winter) with chromium and iron having the largest variations [1, 20]. The variations between the participants cannot be attributed to measurement errors, as the intra‐individual measurements were processed and measured in the same batch. It seems that the personal mineral profile hardly changes unless there is a decisive lifestyle change. We suspect that diet (and the genotype that determines mineral accessibility) is a major driver for changes in the mineral content of fingernails [1].

In general, the average values in our study group matched the mineral contents in fingernails reported in the literature.

A Spearman correlation between the element concentrations in the nails shows some remarkable relations (Figure 1). The alkali metals sodium and potassium show a particularly high positive correlation (*r* = 0.89), whereas the lithium content does not correlate with the other two elements. This correlation was also described by Bahreini et al. and Nowak et al. who found a significant positive correlation of Na and K of *r* = 0.84 (*n* = 27 healthy, 47 osteopenic, and 25 osteoporotic subjects) and *r* = 0.37 (*n* = 64 healthy volunteers), respectively [21, 22]. The relationship between Na and K concentration is also found in serum samples (*r* = 0.21, *n* = 281 postmenopausal women) [23], however it is less pronounced and the amount of sodium ions is 19‐fold the amount of potassium ions. In the nail matrix (keratinocytes) this quotient is 1.4 and indicates a Na^+^/K^+^‐pump activity, similar to other cell types.

The elements calcium and magnesium as well as calcium and phosphorus are also closely related, although magnesium and phosphorus correlate weaker, but still significantly. The literature reveals contradictory results in that both Bahreini et al. and Ohgitani et al. found no correlation between calcium and magnesium, but others found a significant positive correlation (*r* = 0.45, *p* < 0.001) [24] and (*r* = 0.74) [25]. The ratio between calcium and phosphorus concentration in our study is 2.3–1, which roughly reflects the ratio of the two elements in blood plasma [26].

Gutiérrez et al. investigated 14 studies on the correlation of trace elements in toenails and reported strong correlations among iron and cobalt (*r* = 0.81) and manganese and chromium (*r* = 0.72, *p* < 0.001), respectively [25, 27]. We also observed these correlations; however the correlations between the metals manganese and molybdenum, iron and chromium, and iron and cobalt are remarkable and reminiscent of the composition of steels. As in many other studies, stainless steel clippers were used to collect fingernail samples [1], we cannot rule out contamination by metal particles.

For selenium, the strongest correlation was found for sulfur (*r* = 0.36), copper (*r* = 0.29) and zinc (*r* = 0.29). Interestingly, the relation is reflected in various studies that have shown plasma selenium is closely related to copper and zinc plasma levels [28, 29].

In addition to nail and nail bed disorders caused by nutritional deficiencies, trauma, fungi, or tumors, healthy individuals report individual structural characteristics such as brittle fingernails, longitudinal ridges, or white spots [2, 30].

Brittle fingernails have been linked to osteoporosis and disorders of calcium metabolism such as hyperparathyroidism, hypoparathyroidism and malabsorption, respectively [31]. Structural changes can also be observed in diabetes and iron deficiency anemia [1, 2, 5, 32]. Our results indicate a strong negative correlation with potassium, which requires further investigation, as neither element has previously been mentioned in connection with nail diseases or structural characteristics.

The etiology of longitudinal ridges and white dots is not known in detail. Longitudinal ridges (trachyonychia) describe a rough nail surface with gray opacity, longitudinal ridges, and a sandpaper‐like appearance. Trachyonychia is widespread in people over 60 years and has been linked to dehydration due to poor dietary water supply [30]. In our study, we were also able to determine a significant age‐dependent correlation (*p* < 0.001). In addition, we found a significant association with reduced levels of sodium and potassium, two minerals that are directly related to hydration. In contrast, the occurrence of white spots was inversely correlated with age (*p* < 0.001). The significantly lower content of chromium among individuals with white spots cannot be explained yet. In addition, white spots could also occur when the cuticle is forcefully pushed back.

To validate the association of the mineral composition of fingernails with dietary habits and lifestyle factors, we choose selenium since it has been investigated in detail in recent and past studies [11, 28, 33]. The values determined in our study (0.44 ± 0.09 μg/g) confirm the values (toenails) of several studies, e.g., by Gu et al. with vegans (*n* = 76, 0.46 ± 0.11 μg/g), lacto‐ovo vegetarians (*n* = 144, 0.56 ± 0.17 μg/g), vegetarians (*n* = 220, 0.53 ± 0.16 μg/g), and omnivores (*n* = 220, 0.69 ± 0.53 μg/g) [34] or by Chan et al. with pregnant women with gestational diabetes (*n* = 33, 0.53 μg/g) and the control group (*n* = 33, 0.46 μg/g) [4] and values from a Polish study with pooled nail samples from a beauty salon (0.51 ± 0.14 μg/g) [35].

Literature data showed a consistent effect of selenium supplementation and Se content of nails [8, 10]. Since selenium is the most frequently examined element in toe‐ and fingernails, we decided to confirm our results with published studies. As shown above, the selenium content in fingernails from this study is in line with other studies. It is important to note that the selenium content in fingernails is twice as high as in toenails and that selenium levels also vary from country to country. For example, studies from the US and Canada show significantly higher selenium levels than European studies [1]. This is also shown by recent data from the American NHANES study, which found a blood selenium level of 188.7 μg/L and a review on Germans that found a blood selenium level of only 82 μg/L [36, 37]. We found a mean value of 0.44 μg/g (0.22–0.71 μg/g) whereas data from the American population found 0.84 μg/g for female and 0.77 μg/g selenium (in toenail) for male participants [8]. The selenium levels in our study correspond well with the levels found in other European studies (see above).

The strongest influencing factor on selenium levels was the use of selenium supplements. In contrast to Park et al., who found an increase of 6.9%, we see an increase of 21% [8]. This could be due to the different study populations (see above). The American cohort studied by Park et al. shows higher selenium levels, which are presumably only marginally increased by supplementation. In contrast, a Polish cohort shows a significant increase of 20% with supplementation, which is in line with our observations [38].

Diets appear to have a significant influence on the selenium content of fingernails. Selenium species in particular play a decisive role in bioavailability and toxicology [39, 40]. Foodstuff from the questionnaire mainly contains organic selenium species such as selenomethionine and selenocysteine with high bioavailability and comparatively low toxicity. In general, we observe a reduction in selenium content with a decrease in the consumption of animal‐based foods (Figure 2C). This is particularly evident when comparing omnivores and vegans. Due to the high selenium content in fish and seafood, pescatarians have relatively high selenium levels in their nails. This is also confirmed by a recent German study by Klein et al. on vegans and vegetarians. Both the total selenium content in plasma and the enzyme activity of selenium‐dependent glutathione peroxidase (GPX) are significantly reduced in vegans and vegetarians [28]. The study was also confirmed by Simon et al. who investigated 342 children and adolescents with different dietary patterns (86 vegans, 120 vegetarians, 118 omnivores) from the cross‐sectional VeChi Youth study. The authors found lower selenium and zinc but higher copper intake in vegans compared to omnivores [29]. A Chinese study from Shanghai with 220 vegetarians, 76 vegans, 144 lacto‐ovo vegetarians and 220 omnivores found significantly higher toenail selenium in omnivores (vs vegetarians) and lacto‐ovo vegetarians (vs vegans) [34].

It should be noted that selenium has a narrow therapeutic window and supplementation with selenium preparations is only recommended in cases of deficiency. The European Food Safety Agency (EFSA) recommends a tolerable upper intake level of 255 μg/day selenium from any source [41].

Rather unexpectedly, we found significantly higher selenium levels in participants with thyroid disorders. Since the survey did not differentiate between the types of thyroid disorders, we looked at the medication taken by the participants. 74% of those reported a thyroid disorder took medication to treat hypothyroidism whereas 26% did not mention any medication. The thyroid gland contains a high concentration of selenium due to several selenoproteins involved in thyroid hormone metabolism and antioxidant defense, particularly in removing excess hydrogen peroxide during hormone synthesis. Additionally, selenium is crucial for iodothyronine deiodinases that convert T4 to T3, but this effect appears unaffected in humans under normal selenium deficiency due to the high priority of these enzymes in selenium distribution [42]. However, a study by Winther et al. found reduced TSH and T4 plasma levels when selenium was supplemented in euthyroid patients and did not recommend selenium supplementation under conditions of marginal Se deficiency [43]. Although we did not record any clinical data, such as thyroid hormone levels, we assume that the result is biased by a selenium‐rich diet, such as sea food which is recommended by several health associations. Future studies should also measure thyroid hormone levels.

Although other studies sporadically identified correlations with demographic factors [44], age [45], gender [10], tobacco [38] and alcohol consumption [46], respectively, we cannot confirm these findings in our study.

Half of the studies that have measured selenium levels in toenails show a direct correlation between selenium levels and dietary selenium intake. Especially the consumption of breads [8], beef [8], eggs and dairy products positively correlates with the selenium in toenails [1]. In German food, the highest levels of selenium were found in porcini mushroom (*boletus edulis*) (3.13 mg/kg), seafood (0.31 mg/kg), eggs and egg products (0.22 mg/kg) and meat (0.2 mg/kg) [47]. This is in agreement with our descriptive observation that regular consumption of fish and seafood, eggs and meat may be associated with higher selenium concentrations. In these food groups, selenium is present in the form of selenomethionine or selenocysteine, which are both highly bioavailable. Thus, the difference in selenium levels in fingernails found between omnivores and vegans in our study seems plausible.

We identified several limitations of the study. Our study is not representative, as can be seen from the age and gender distribution. In addition, the proportion of vegetarians and vegans is disproportionately high. Our questionnaire recorded a semi‐quantitative food frequency that was not validated. In addition, it was not possible to derive numerical values using a food code or index. Our analyses were explorative, and further multivariable analyses are needed to draw stronger conclusions on potential associations between diet, lifestyle and medical parameters and mineral concentrations. A comparison of biomarkers of selenium status (serum selenium content, selenoprotein P, GPX activity.) would have facilitated the evaluation of the fingernail data. Thus, we cannot conclude the selenium status based on the fingernail content. In addition to the minerals listed, it would have been interesting to determine toxic elements such as cadmium, arsenic or lead. The determination of iodine should also be integrated into future studies.

## 5 Conclusions

The Fulda NutriNAIL study aims to combine bibliographic data with mineral analyses and fingernail surface patterns. Our study provides robust evidence that fingernail mineral profiles reflect both dietary habits and supplement use, with selenium emerging as a particularly responsive marker. While intra‐individual mineral levels remained relatively stable over time, considerable inter‐individual variation was observed, likely driven by lifestyle factors such as diet and supplementation patterns. Notably, selenium levels were significantly associated with dietary patterns—particularly the degree of animal‐based food intake—and the use of supplements, with omnivores showing higher concentrations compared to vegans. Elevated selenium levels in participants with thyroid disorders may reflect targeted dietary recommendations or unknown underlying metabolic changes. The correlations between specific minerals (e.g., Na–K, Ca–Mg, Fe–Co) underscore the potential of fingernail analysis as a non‐invasive biomarker tool for future studies. These should aim to include toxic elements, iodine, and validated biomarkers of mineral status to better contextualize and validate fingernail‐based assessments.

## Author Contributions

**Marc Birringer**, **Nina Sonntag**, and **Jan‐Torsten Milde:** conceptualization. **Tilman Kühn**, **Alexander Maxones**, **Nina Sonntag**, **Katja Plendl**, **Lina Müller**, **Dustin Dewald**, and **Marc Birringer:** methodology. **Marc Birringer**, **Alexander Maxones**, **Katja Plendl**, **Lina Müller**, and **Tilman Kühn:** validation. **Heike Hollenbach**, **Alexander Maxones**, **Tilman Kühn**, **Nina Sonntag**, and **Marc Birringer:** formal analysis. **Marc Birringer:** writing – original draft preparation. **Tilman Kühn**, **Alexander Maxones**, **Nina Sonntag**, **Katja Plendl**, **Lina Müller**, and **Marc Birringer:** writing – review and editing. **Marc Birringer:** supervision. **Marc Birringer:** project administration. All authors have read and agreed to the published version of the manuscript.

## Conflicts of Interest

The authors declare no conflicts of interest.

## Supporting information


**Table S1:** Self‐reported dietary habits of study participants and Se‐content (μg/g) of nails.

**Data S1:** Supporting Information.

## Data Availability

The data that support the findings of this study are available from the corresponding author upon reasonable request.
