# Supplementary material for: Fingernail Mineral Profiling as a Non‐Invasive Tool to Assess Dietary and Lifestyle Factors: Results From the Cross‐Sectional Fulda NutriNAIL Study
Source: Biofactors. 2025 Nov 14;51(6):e70056. doi: 10.1002/biof.70056 (PMC12616768; doi:10.1002/biof.70056)
Supplement: Supplementary file 1 — Table S1: Self‐reported dietary habits of study participants and Se‐content (μg/g) of nails. [file BIOF-51-0-s002.docx]

Table S1: Self-reported dietary habits of study participants and Se-content (µg/g) of nails

|  |  | **selenium (µg/g)** | |  |  | **selenium (µg/g)** | |
| --- | --- | --- | --- | --- | --- | --- | --- |
| **consumption** | **frequency** | **mean (n)** | **(min-max)** | **consumption** | **frequency** | **mean (n)** | **(min-max)** |
| sweets | daily | 0,43 (53) | 0,22-0,70 | dried fruit | daily | 0,43 (10) | 0,33-0,70 |
|  | weekly | 0,44 (78) | 0,27-0,71 |  | weekly | 0,44 (22) | 0,27-0,71 |
|  | several times a month | 0,40 (26) | 0,24-0,64 |  | several times a month | 0,41 (49) | 0,24-0,64 |
|  | rarely | 0,47 (5) | 0,39-0,55 |  | rarely | 0,43 (52) | 0,22-0,65 |
|  | never | 0,32 (2) | 0,31-0,32 |  | never | 0,43 (30) | 0,28-0,56 |
| snacks | daily | 0,45 (5) | 0,32-0,59 |  | don't know | 0,53 (1) | 0,53-0,53 |
|  | weekly | 0,44 (63) | 0,27-0,70 | cabbage | daily | 0,43 (4) | 0,35-0,51 |
|  | several times a month | 0,41 (56) | 0,22-0,65 |  | weekly | 0,43 (72) | 0,27-0,71 |
|  | rarely | 0,45 (29) | 0,32-0,71 |  | several times a month | 0,43 (70) | 0,22-0,70 |
|  | never | 0,38 (11) | 0,31-0,45 |  | rarely | 0,44 (16) | 0,32-0,54 |
| ready-made products | daily | 0,59 (1) | 0,59-0,59 |  | never | 0,49 (2) | 0,42-0,56 |
|  | weekly | 0,42 (41) | 0,22-0,70 | sea fish and maritime products | daily | 0,31 (2) | 0,28-0,35 |
|  | several times a month | 0,42 (62) | 0,24-0,63 |  | weekly | 0,45 (27) | 0,32-0,71 |
|  | rarely | 0,45 (47) | 0,31-0,71 |  | several times a month | 0,44 (58) | 0,22-0,70 |
|  | never | 0,43 (13) | 0,31-0,52 |  | rarely | 0,43 (35) | 0,28-0,62 |
| vegan/vegetarian substitute products | daily | 0,39 (17) | 0,27-0,50 |  | never | 0,41 (42) | 0,24-0,63 |
|  | weekly | 0,41 (37) | 0,22-0,55 | green leafy vegetables | daily | 0,44 (19) | 0,32-0,71 |
|  | several times a month | 0,46 (29) | 0,34-0,70 |  | weekly | 0,43 (116) | 0,22-0,70 |
|  | rarely | 0,44 (29) | 0,28-0,65 |  | several times a month | 0,43 (25) | 0,28-0,56 |
|  | never | 0,42 (52) | 0,24-0,71 |  | rarely | 0,42 (3) | 0,28-0,53 |
| fruit | daily | 0,42 (93) | 0,22-0,71 |  | never | 0,27 (1) | 0,27-0,27 |
|  | weekly | 0,44 (57) | 0,32-0,65 | root vegetables | daily | 0,39 (14) | 0,29-0,52 |
|  | several times a month | 0,44 (14) | 0,32-0,59 |  | weekly | 0,43 (131) | 0,22-0,71 |
|  | rarely | - | - |  | several times a month | 0,42 (15) | 0,24-0,52 |
|  | never | - | - |  | rarely | 0,45 (4) | 0,35-0,54 |
| vegetables | daily | 0,42 (115) | 0,22-0,71 |  | never | - | - |
|  | weekly | 0,44 (48) | 0,32-0,65 | rice | daily | 0,46 (3) | 0,39-0,51 |
|  | several times a month | 0,45 (1) | 0,45-0,45 |  | weekly | 0,43 (74) | 0,28-0,66 |
|  | rarely | - | - |  | several times a month | 0,43 (70) | 0,22-0,71 |
|  | never | - | - |  | rarely | 0,42 (17) | 0,31-0,54 |
| milk and dairy products | daily | 0,44 (87) | 0,24-0,71 |  | never | - | - |
|  | weekly | 0,42 (55) | 0,22-0,70 | beer and mixed beer drinks | daily | 0,34 (1) | 0,34-0,34 |
|  | several times a month | 0,42 (8) | 0,34-0,54 |  | weekly | 0,43 (28) | 0,31-0,70 |
|  | rarely | 0,39 (3) | 0,33-0,48 |  | several times a month | 0,44 (51) | 0,22-0,66 |
|  | never | 0,40 (11) | 0,27-0,55 |  | rarely | 0,42 (44) | 0,27-0,64 |
| fortified vegan/vegetarian substitute products | daily | 0,41 (24) | 0,22-0,70 |  | never | 0,42 (39) | 0,24-0,71 |
|  | weekly | 0,42 (36) | 0,27-0,63 |  | don't know | 0,53 (1) | 0,53-0,53 |
|  | several times a month | 0,44 (22) | 0,32-0,62 | wine and mixed wine drinks | daily | 0,33 (1) | 0,33-0,33 |
|  | rarely | 0,48 (22) | 0,33-0,65 |  | weekly | 0,45 (28) | 0,32-0,63 |
|  | never | 0,42 (53) | 0,24-0,71 |  | several times a month | 0,43 (56) | 0,22-0,70 |
|  | don't know | 0,47 (7) | 0,35-0,66 |  | rarely | 0,43 (40) | 0,27-0,55 |
| eggs | daily | 0,42 (6) | 0,29-0,62 |  | never | 0,42 (39) | 0,24-0,71 |
|  | weekly | 0,44 (83) | 0,31-0,71 | spirits | daily | - | - |
|  | several times a month | 0,43 (52) | 0,22-0,70 |  | weekly | 0,41 (10) | 0,31-0,52 |
|  | rarely | 0,41 (12) | 0,28-0,54 |  | several times a month | 0,44 (44) | 0,31-0,64 |
|  | never | 0,38 (11) | 0,27-0,55 |  | rarely | 0,43 (59) | 0,22-0,70 |
| meat and sausage | daily | 0,40 (22) | 0,28-0,54 |  | never | 0,42 (50) | 0,24-0,71 |
|  | weekly | 0,45 (61) | 0,22-0,66 |  | don't know | 0,53 (1) | 0,53-0,53 |
|  | several times a month | 0,43 (23) | 0,28-0,71 | **purchase of organic food** | almost ever | 0,46 (25) | 0,24-0,71 |
|  | rarely | 0,46 (11) | 0,34-0,70 |  | frequently | 0,41 (86) | 0,22-0,66 |
|  | never | 0,41 (47) | 0,24-0,63 |  | rarely | 0,45 (53) | 0,28-0,70 |
| offal and products made from it | daily | 0,36 (1) | 0,36-0,36 |  | never | - | - |
|  | weekly | 0,36 (3) | 0,32-0,39 | **salt use** | non-fortified salt | 0,41 (23) | 0,27-0,55 |
|  | several times a month | 0,44 (20) | 0,29-0,62 |  | iodized table salt | 0,43 (93) | 0,22-0,71 |
|  | rarely | 0,44 (50) | 0,28-0,71 |  | sea salt | 0,42 (27) | 0,31-0,62 |
|  | never | 0,42 (88) | 0,22-0,70 |  | iodized sea salt | 0,48 (12) | 0,29-0,64 |
|  | don't know | 0,49 (2) | 0,39-0,58 |  | low-sodium salt (potassium salt) | - | - |
| whole grain products | daily | 0,43 (80) | 0,24-0,71 |  | none | 0,24 (1) | 0,24-0,24 |
|  | weekly | 0,43 (72) | 0,22-0,66 | **main source of hydration** |  |  |  |
|  | several times a month | 0,44 (11) | 0,33-0,53 | commercial mineral water | not selected | 0,42 (107) | 0,22-0,70 |
|  | rarely | 0,40 (1) | 0,40-0,40 |  | selected | 0,44 (57) | 0,24-0,71 |
|  | never | - | - | tap water | not selected | 0,44 (37) | 0,24-0,71 |
| nuts | daily | 0,42 (36) | 0,24-0,70 |  | selected | 0,43 (127) | 0,22-0,70 |
|  | weekly | 0,43 (79) | 0,22-0,71 | coffee | not selected | 0,43 (111) | 0,22-0,71 |
|  | several times a month | 0,46 (36) | 0,32-0,65 |  | selected | 0,42 (53) | 0,27-0,63 |
|  | rarely | 0,38 (10) | 0,28-0,52 | tea | not selected | 0,43 (107) | 0,22-0,70 |
|  | never | 0,38 (3) | 0,33-0,44 |  | selected | 0,43 (57) | 0,27-0,71 |
| pulses | daily | 0,39 (15) | 0,24-0,50 | sweetened beverages | not selected | 0,43 (154) | 0,22-0,71 |
|  | weekly | 0,43 (92) | 0,22-0,71 |  | selected | 0,42 (10) | 0,28-0,56 |
|  | several times a month | 0,43 (40) | 0,29-0,63 | **selenium supplement use** | not selected | 0,42 (164) | 0,22-0,71 |
|  | rarely | 0,42 (14) | 0,32-0,56 |  | selected | 0,51 (20) | 0,34-0,70 |
|  | never | 0,47 (1) | 0,47-0,47 |  |  |  |  |
|  | don't know | 0,43 (2) | 0,38-0,47 |  |  |  |  |
