# Supplementary material for: Fingernail Mineral Profiling as a Non‐Invasive Tool to Assess Dietary and Lifestyle Factors: Results From the Cross‐Sectional Fulda NutriNAIL Study
Source: Biofactors. 2025 Nov 14;51(6):e70056. doi: 10.1002/biof.70056 (PMC12616768; doi:10.1002/biof.70056)
Supplement: Supplementary file 2 — Data S1: Supporting Information. [file BIOF-51-0-s001.pdf]

QR01

Paste your copied QR code here:

Code must be 8 digits

QR code: Code must be 8 digits

EV08

Dear study participants,

welcome to the NutriNAIL study survey.

In addition to taking photos of your fingernails and providing fingernail samples, we would like to ask you to answer the following questions about your diet, health and lifestyle. This is the only way we can achieve meaningful study results.

The survey consists of a total of 24 questions. We ask you to answer the questions carefully and honestly. Your information will be treated anonymously and confidentially and used exclusively for scientific purposes. Your answers are subject to data protection regulations. Accordingly, your answers cannot be traced back to you.

You can answer most questions by clicking on the appropriate answer. If multiple answers or text input is required for your answer, this is noted under the question. If you have problems filling out the questionnaire, it is best to ask the staff present in the study room.

Thank you for taking the time to take part in our survey. Your opinion and your information are very important to us!

Your NutriNAIL team at Fulda University of Applied Sciences

EV01

What diet do you mainly follow?

- ○ Omnivorous
(All Food)
- ○ Pescetarian
(Fish, eggs, milk/milk products, plant-based foods)
- ○ Ovo-Lacto-Vegetarian
(eggs, milk/milk products, plant-based foods)
- ○ Lacto-Vegetarian
(Milk/milk products, plant-based foods)
- ○ Ovo-Vegetarian
(eggs, plant-based foods)
- ○ Vegan
(purely plant-based food)
- ○ Other: __________

EV05

How often do you consume the following foods in your daily diet?

Select the answer that best applies to you in each line.

Portion of fruit
(1 portion = 1 palm size)

Several times a day	Once a day	4-6 times per week	1-3 times per week	1-3 times per month	Less than once per month	Never	I do not know

Portion of vegetables
(1 portion = 1 palm size)

Several times a day	Once a day	4-6 times per week	1-3 times per week	1-3 times per month	Less than once per month	Never	I do not know

Milk and dairy products (e.g. cheese, yoghurt, etc.)

Several times a day	Once a day	4-6 times per week	1-3 times per week	1-3 times per month	Less than once per month	Never	I do not know

Enriched, vegan/vegetarian substitute products (e.g. plant drinks with calcium, meat substitutes with iron)

Several times a day	Once a day	4-6 times per week	1-3 times per week	1-3 times per month	Less than once per month	Never	I do not know

Eggs

Several times a day	Once a day	4-6 times per week	1-3 times per week	1-3 times per month	Less than once per month	Never	I do not know

Meat and sausages

Several times a day	Once a day	4-6 times per week	1-3 times per week	1-3 times per month	Less than once per month	Never	I do not know

Offal and products thereof (e.g. liver sausage)

Several times a day	Once a day	4-6 times per week	1-3 times per week	1-3 times per month	Less than once per month	Never	I do not know

Whole grain products (e.g. whole grain bread, oat flakes, etc.)

Several times a day	Once a day	4-6 times per week	1-3 times per week	1-3 times per month	Less than once per month	Never	I do not know

Nuts (unprocessed or ground form)

Several times a day	Once a day	4-6 times per week	1-3 times per week	1-3 times per month	Less than once per month	Never	I do not know

Legumes (e.g. beans, peas, lentils, soya/soy products, etc.)

Several times a day	Once a day	4-6 times per week	1-3 times per week	1-3 times per month	Less than once per month	Never	I do not know

Dried fruits

Several times a day	Once a day	4-6 times per week	1-3 times per week	1-3 times per month	Less than once per month	Never	I do not know

Brassica vegetables (e.g. broccoli, white cabbage, etc.)

Several times a day	Once a day	4-6 times per week	1-3 times per week	1-3 times per month	Less than once per month	Never	I do not know

Sea fish and maritime products (e.g. tuna, seafood, seaweed, etc.)

Several times a day	Once a day	4-6 times per week	1-3 times per week	1-3 times per month	Less than once per month	Never	I do not know

Green leafy vegetables (e.g. spinach, kale, lettuce, etc.)

Several times a day	Once a day	4-6 times per week	1-3 times per week	1-3 times per month	Less than once per month	Never	I do not know

Root vegetables (e.g. carrots, potatoes, beetroot, etc.)

Several times a day	Once a day	4-6 times per week	1-3 times per week	1-3 times per month	Less than once per month	Never	I do not know

rice

Several times a day	Once a day	4-6 times per week	1-3 times per week	1-3 times per month	Less than once per month	Never	I do not know

Beer and mixed drinks
(excluding non-alcoholic beer)

Several times a day	Once a day	4-6 times per week	1-3 times per week	1-3 times per month	Less than once per month	Never	I do not know

Wine and mixed drinks

Several times a day	Once a day	4-6 times per week	1-3 times per week	1-3 times per month	Less than once per month	Never	I do not know

Spirits and mixed drinks

Several times a day	Once a day	4-6 times per week	1-3 times per week	1-3 times per month	Less than once per month	Never	I do not know

EV02

How often do you consume foods that are high in sugar or processed?

Select the answer that best applies to you in each line.

sweets

Several times a day	Once a day	4-6 times per week	1-3 times per week	1-3 times per month	Less than once a month	Never

Snacks

Several times a day	Once a day	4-6 times per week	1-3 times per week	1-3 times per month	Less than once a month	Never

Ready-made products/meals (e.g. sauces, frozen/canned meals, etc.)

Several times a day	Once a day	4-6 times per week	1-3 times per week	1-3 times per month	Less than once a month	Never

Vegan/vegetarian substitute products without enrichment (e.g. egg substitutes, pure plant drinks/yoghurts, etc.)

Several times a day	Once a day	4-6 times per week	1-3 times per week	1-3 times per month	Less than once a month	Never

EV06

How often do you buy organic products?

- (Almost always
- Frequently
- Rarely
- Never

EV03

Which table salt do you mainly use?

- Normal salt (without additives)
- Iodized salt
- sea-salt
- Iodized sea salt
- Low sodium salt (potassium salt)
- I don’t use salt

- I do not know

EV04

How do you primarily meet your water needs?

- ☐ Purchased mineral water
- ☐ tap water
- ☐ Coffee
- ☐ tea
- ☐ sweetened drinks
  (e.g. lemonade, iced tea, energy drinks, etc.)

EV07

Are you currently taking or have you taken any dietary supplements in the last 12 months?

(Multiple selection possible)

- ☐ Biotin
- ☐ Calcium
- ☐ iron
- ☐ iodine
- ☐ magnesium
- ☐ selenium
- ☐ zinc
- ☐ Vitamin B12
- ☐ Other B vitamins
  Optionally, enter the supplemented B vitamins here.
- ☐ Vitamin D
- ☐ Miscellaneous:

- ☐ I do not know
- ☐ No

GS01

How tall are you?

Size (in cm:

GS02

What’s your weight?

Weight in kg: ____________________

GS03

Have you ever been diagnosed with any of the following diseases/syndromes by a doctor?

(Multiple selection possible)

- ☐ high blood pressure
- ☐ Heart attack
- ☐ stroke
- ☐ Stomach/intestinal ulcer
- ☐ Chronic intestinal diseases (Crohn’s disease, ulcerative colitis)
- ☐ Diabetes mellitus type 1
- ☐ Diabetes mellitus type 2
- ☐ High cholesterol or fat levels in the blood
- ☐ Gout/increased uric acid
- ☐ osteoporosis
- ☐ asthma
- ☐ Allergic rhinitis, hay fever, contact allergy
- ☐ Neurodermatitis
- ☐ psoriasis
- ☐ Food allergy

  Please enter your allergy(ies) here. E.g. celery, nuts, milk, ...

- [ ] Food intolerance

  Please enter your intolerance(s) here. E.g. lactose, fructose, gluten, ...
- [ ] Thyroid disease
- [ ] immunodeficiency
- [ ] Addiction
- [ ] Chronic kidney disease
- [ ] Chronic liver disease
- [ ] Joint disease (arthritis, arthrosis, rheumatism)
- [ ] Other autoimmune diseases (e.g. multiple sclerosis)
- [ ] Eating disorders (e.g. bulimia, anorexia)
- [ ] Other:

- [ ] No

GS06

Do you take any medication regularly? If so, which one (name of medication)?

(Multiple entries possible, separated by commas)

- ○ Yes, namely:
- ○ No

GS04

Have you ever been diagnosed with a nutrient deficiency (e.g. iron deficiency)?

(Multiple entries possible, separated by commas)

- ○ Yes, namely: ____________
- ○ No

- ○ Weiß nicht

1 Active Filter(s)

Filter GS04/F1
If any of the following options is selected: **1**
Then display question/text **GS05** placed later in the questionnaire (otherwise hide)

GS05

Does this nutrient deficiency currently exist?

- ○ Yes
- ○ Yes, several: ____________
- ○ No

- ○ Weiß nicht

LS01

Describe your daily activity level:

(Sports units excluded)

- ◯ Exclusively sedentary or lying lifestyle
- ◯ Exclusively sedentary work with little or no other strenuous activities
- ◯ Sedentary activity, occasionally additional energy expenditure for walking or standing activity
- ◯ Mainly walking and standing work
- ◯ Physically demanding professional work

LS02

How often do you exercise per week?

(at least 30 minutes, high intensity = continuous conversation no longer possible)

- ◯ 5 times or more
- ◯ 3–4 times
- ◯ 1–2 times
- ◯ Never

1 Active Filter(s)

Filter LS02/F1
If any of the following options is selected: **1, 2, 3**
Then display question/text **LS03** placed later in the questionnaire (otherwise hide)

LS03

Where do you mainly carry out your sports sessions?

- ◯ Outdoors
- ◯ Indoors
- ◯ 50% outdoors, 50% indoors

LS05

How often do you smoke the following products?

Select the answer that best applies to you in each line.

	Several times a day	Once a day	4-6 times per week	1-3 times per week	1-3 times per month	Less than once per month	Never
Cigarettes	◯	◯	◯	◯	◯	◯	◯
E-cigarettes	◯	◯	◯	◯	◯	◯	◯
Other tobacco products (e.g. snuff/chewing tobacco)	◯	◯	◯	◯	◯	◯	◯
cannabis	◯	◯	◯	◯	◯	◯	◯

LS06

Do you often have problems with your fingernails or have you noticed visual changes to your nails?

- ◯ Brittle
- ◯ Thin
- ◯ White dots
- ◯ Discoloration
- ◯ Longitudinal corrugation
- ◯ Miscellaneous: __________

- ◯ No

LS07

How often do you cut or care for your fingernails?

- ◯ Daily
- ◯ Weekly
- ◯ Monthly
- ◯ Less common

LS08

How often do you paint your nails and/or use nail (care) products?

- ◯ Always
- ◯ Sometimes
- ◯ Rarely
- ◯ Never

1 Active Filter(s)

Filter LS08/F1
If any of the following options is selected: **1, 2, 3**
Then display question/text **LS09** placed later in the questionnaire (otherwise hide)

LS09

Have you used silver, gold, copper or bronze nail polish in the last six months?

- ◯ Yes, color: __________
- ◯ No

SD01

What is your biological sex?

- ◯ female
- ◯ masculine

SD02

How old are you?

I am ____ Years

SD10

What is the highest level of education you have?

- ◯ Secondary school certificate/elementary school certificate or lower
- ◯ Secondary school / high school)
- ◯ (Technical) high school diploma, general or subject-specific university entrance qualification
- ◯ Bachelor
- ◯ Master/PhD
- ◯ Completed training/master
- ◯ Other school qualifications: ____

SD14

What is your relationship to Fulda University?

- ◯ Student
- ◯ Colleague
- ◯ External

SD18

Would you like to add anything to this survey or to help us better understand your answers?

Did you notice anything negative while taking part in this survey? Were the questions unclear at some point or did you find it uncomfortable to answer them? Please write a few key words about it.

SD19

Thank you very much for taking the time to fill out our questionnaire and take part in the NutriNAIL study.

As a small thank you for your support, if you gave your consent when registering, you have automatically qualified for our competition to win a €50 cafeteria voucher. We'll keep our fingers crossed for you and wish you the best of luck!

The winners will be informed by email after the study has been completed. So keep an eye on your inbox!

Thank you again for your participation and commitment. If you have any questions, we will be happy to help. Best wishes, your NutriNAIL team at Fulda University of Applied Sciences

Your answers have been saved, you can now close the browser window.
